# Apparent Saturation of Branched-Chain Amino Acid Catabolism After High Dietary Milk Protein Intake in Healthy Adults

**DOI:** 10.1210/clinem/dgae599

**Published:** 2024-09-20

**Authors:** Emily Newton-Tanzer, Sultan Nilay Can, Hans Demmelmair, Jeannie Horak, Lesca Holdt, Berthold Koletzko, Veit Grote

**Affiliations:** Division of Metabolic and Nutritional Medicine, Department Paediatrics, Dr. von Hauner Children's Hospital, LMU University Hospital, LMU Munich, and the German Center for Child and Adolescent Health, site Munich, 80337 Munich, Germany; Division of Metabolic and Nutritional Medicine, Department Paediatrics, Dr. von Hauner Children's Hospital, LMU University Hospital, LMU Munich, and the German Center for Child and Adolescent Health, site Munich, 80337 Munich, Germany; Division of Metabolic and Nutritional Medicine, Department Paediatrics, Dr. von Hauner Children's Hospital, LMU University Hospital, LMU Munich, and the German Center for Child and Adolescent Health, site Munich, 80337 Munich, Germany; Division of Metabolic and Nutritional Medicine, Department Paediatrics, Dr. von Hauner Children's Hospital, LMU University Hospital, LMU Munich, and the German Center for Child and Adolescent Health, site Munich, 80337 Munich, Germany; Institute of Laboratory Medicine, LMU University Hospital, LMU Munich, 80337 Munich, Germany; Division of Metabolic and Nutritional Medicine, Department Paediatrics, Dr. von Hauner Children's Hospital, LMU University Hospital, LMU Munich, and the German Center for Child and Adolescent Health, site Munich, 80337 Munich, Germany; Division of Metabolic and Nutritional Medicine, Department Paediatrics, Dr. von Hauner Children's Hospital, LMU University Hospital, LMU Munich, and the German Center for Child and Adolescent Health, site Munich, 80337 Munich, Germany

**Keywords:** young child formulas, milk protein, diet, branched-chain amino acids, acylcarnitine, branched-chain ketoacids, postprandial phase, the early protein hypothesis

## Abstract

**Context:**

Milk protein contains high concentrations of branched-chain amino acids (BCAA) that play a critical role in anabolism and are implicated in the onset of obesity and chronic disease. Characterizing BCAA catabolism in the postprandial phase could elucidate the impact of protein intake on obesity risk established in the “early protein hypothesis.”

**Objective:**

To examine the acute effects of protein content of young child formulas as test meals on BCAA catabolism, observing postprandial plasma concentrations of BCAA in relation to their degradation products.

**Methods:**

The TOMI Add-On Study is a randomized, double-blind crossover study in which 27 healthy adults consumed 2 isocaloric young child formulas with alternating higher (HP) and lower (LP) protein and fat content as test meals during separate interventions, while 9 blood samples were obtained over 5 hours. BCAA, branched-chain α-keto acids (BCKA), and acylcarnitines were analyzed using a fully targeted HPLC-ESI-MS/MS approach.

**Results:**

Mean concentrations of BCAA, BCKA, and acylcarnitines were significantly higher after HP than LP over the 5 postprandial hours, except for the BCKA α-ketoisovalerate (KIVA). The latter metabolite showed higher postprandial concentrations after LP. With increasing mean concentrations of BCAA, concentrations of corresponding BCKA, acylcarnitines, and urea increased until a breakpoint was reached, after which concentrations of degradation products decreased (for all metabolites except valine and KIVA and Carn C4:0-iso).

**Conclusion:**

BCAA catabolism is markedly influenced by protein content of the test meal. We present novel evidence for the apparent saturation of the BCAA degradation pathway in the acute postprandial phase up to 5 hours after consumption.

The early protein hypothesis suggests that higher milk protein in infancy increases weight gain, as well as the subsequent risk for obesity in later life (1). This hypothesis has been strongly supported by pediatric observational studies, suggesting the benefits of lower protein intake in infancy and toddlerhood against an increased risk for obesity and associated diseases (2, 3). In particular, the Childhood Obesity Project (CHOP) (4) trial depicted that higher protein intake with infant formula compared to human milk induces higher blood levels of IGF-1, insulin, and plasma concentrations of branched-chain amino acids (BCAA) (5, 6) and serum urea (7, 8). These effects have been associated with the fact that milk protein is high in BCAA, which have been shown to possess insulinogenic properties (9-11).

Research has also suggested that these effects are not necessarily restricted to infancy but can also impact later child development and adulthood. The Toddler Milk Intervention Study trial (TOMI, clinicaltrial.gov: NCT02907502), a double-blind randomized trial, was thus initiated to observe the effects of 2 isocaloric young child formulas with alternating higher (HP) and lower protein (LP) and fat content on toddler growth and metabolism (12). However, as sequential blood sampling is not feasible in infants and/or small children, TOMI cannot provide insight into the short-term postprandial effects of the 2 young child formulas. Therefore, in the TOMI Add-On Study, the young child formulas from the TOMI trial were utilized as test meals in young adult volunteers. Initial results indicated that the increased milk protein supply induced increases in postprandial BCAA concentrations for the entirety of the study, and lead to higher initial insulin secretion (13). These elevated concentrations of BCAA up to 5 hours postprandially draw specific attention to BCAA catabolization, emphasizing that concentrations even remain elevated after the postprandial phase has ended. In addition, results from adult studies proposed circulating BCAA as potential biomarkers for the development of obesity-associated insulin resistance (14) and diabetes (15). Further, increased concentrations of acylcarnitines were noted in study populations of obese adults (16), so that the incomplete oxidation of BCAA during catabolism may contribute to mitochondrial misfunction in diabetes support through anaplerotic stress, as well as an anaplerosis-cataplerosis imbalance (17). To better elucidate these questions, an analysis of samples from the CHOP trial also hypothesized that this increase in BCAA concentrations after high protein intake may be due to the oversaturation of the BCAA decarboxylation pathway, contributing to the anabolic effects of a higher protein diet and the subsequent increased risk for obesity and adiposity (18). However, as these results were established from samples in a steady state during a long-term dietary intervention, it was unclear whether this effect could also be observed in the acute, postprandial phase.

The BCAA decarboxylation pathway is also of particular relevance, as BCAA represent a distinct subtype of indispensable amino acids with important roles in both protein synthesis and the suppression of proteolysis (19), as well as the regulation of body fat deposition through signaling of the mammalian target of rapamycin complex 1 (mTORC1) (20). Both BCAA and their degradation products, branched-chain α-ketoacids (BCKA) can activate mTORC1 (14, 21), stimulating insulin resistance and impaired glucose metabolism (14, 22-24). Plasma BCAA concentrations in both steady-state and postprandial conditions are affected by diet (14, 16, 25), and in particular by the amount and type of protein consumed (26, 27). Further, both BCAA, as well as their catabolites (BCKA and short-chain acylcarnitines) were found elevated in chronic conditions, such as obesity and type 2 diabetes (16, 28, 29), and were shown to be predictive markers for future risk of acquiring these conditions (30-32).

The BCAA decarboxylation pathway, (depicted in supplemental material, Figure S1: https://doi.org/10.6084/m9.figshare.26202359.v1), is made up of 2 predominant steps (33). First, reversible transamination is catalyzed by mitochondrial branched-chain aminotransferase (BCAT), which yields the branched-chain ketoacids (BCKA): α-ketoisocaproate/4-methyl-2-oxovaleric acid (KICA) from leucine, α-keto-β-methylvalerate/3-methyl-2-oxovaleric acid (KMVA) from isoleucine, and α-ketoisovalerate/3-methyl-2-oxobutanoic acid (KIVA) from valine. The second, rate-limiting and irreversible oxidative decarboxylation is catalyzed by the branched-chain alpha-ketoacid dehydrogenase complex (BCKDH). After this step, each acyl-CoA is catabolized according to its own pathway, ultimately yielding acetyl-CoA and/or propionyl-CoA that can then enter the citric acid cycle, act as a precursor for gluconeogenesis, or when in excess can contribute to lipogenesis (34). Acylcarnitines are acyl esters of carnitine that can also arise from the catabolization of BCAA into acylcarnitines (35). Through the generation of acetyl-CoA, leucine generates Carn C5:0-3M, isoleucine generates Carn C5:0-2M, and valine generates Carn C4:0-iso (14, 36). It should be noted that in case of the acylcarnitine terminology, *Carn C5:0-2M* and *Carn C5:0-3M* stand for the position of the methyl side group, while *iso* stands for an iso-methyl branching.

In the present paper, we examined plasma samples collected during the TOMI Add-On Study, focusing predominantly on BCAA catabolization in the acute, postprandial state. As little research focuses on the specific effects of a controlled meal on amino acid degradation in the postprandial phase, these samples offered the unique opportunity to compare the breakdown of BCAA after higher and lower protein intake. We hypothesized that the consumption of HP would oversaturate the BCAA decarboxylation pathway in the acute metabolism.

## Methods

### Study Design and Subjects

Study subjects and design with an accompanying CONSORT diagram have been described in detail in Newton-Tanzer et al (13). The trial was a double-blind, randomized, crossover trial performed at the Dr. von Hauner Children's Hospital at LMU University Munich in 27 healthy, young adults. The study was composed of 2 separate interventions in randomized order, approximately a week apart, where subjects consumed 2 standardized test meals. Between tests, subjects were instructed not to change any major aspects of their lifestyles (diet, physical activity, alcohol consumption), and participants were asked to fast for 12 hours before arriving at the study site for each intervention. Each test meal (HP or LP) was composed of isocaloric young child formulas, with adaptation of the content of fat with equal composition to achieve equal energy density. These formulas were initially created for the Toddler Milk Intervention Study-TOMI trial (clinicaltrial.gov: NCT02907502) (12). HP contained 2010 KJ/480 kcal energy, 30 g protein, 67 g carbohydrates and 10 g fat per 500 mL, while LP contained 2010 KJ/480 kcal energy, 7 g protein, 67 g carbohydrates and 20 g fat per 500 mL, representing a mean per body weight intake of 0.46± 0.09 g/kg and 0.11± 0.02 g/kg, respectively.

Before the first intervention, an informed consent form, and a health and well-being questionnaire was completed. Subjects were asked to fast for 12 hours before arriving at the study site at 8:00 Am, upon which anthropometric measurements were taken (height, weight, and waist circumference). Body fat content and percentage were then assessed via air displacement plethysmography (BOD POD©, COSMED, Fridolfing, Germany), and body mass index (BMI) was calculated. Just before the intervention, a venous catheter was inserted, and initial baseline blood samples were taken. Participants then consumed the test meal within 5 minutes while sitting down. Timing for subsequent blood samples began once the test meal was completely consumed (during this 5-minute period) and venous blood samples were taken at 15, 30, 60, 90, 120, 180, 240, and 300 minutes postprandial to measure various metabolites.

### Laboratory Measurements

In addition to the sample preparation and amino acid analysis discussed in Newton-Tanzer et al (13), organic acids, including ketoacids (37), as well as acylcarnitines (38, 39) were analyzed using a fully targeted high-performance liquid chromatography-electrospray ionization tandem mass spectrometry (HPLC-ESI-MS/MS) approach. For amino acid analysis (13) on an Xbridge C18 column (150 mm × 2.1 mm, 3.5 µm; 186003023) from Waters, an Agilent 1100 HPLC system consisting of a binary pump (G1312A), degasser (G1379A), column oven (G1316A) and a temperature controlled wellplate autosampler (G1367A with G1330B) in combination with an API 2000 triple quadrupole mass spectrometer from Sciex was used. Organic acid analysis (37) was performed on a Kinetex F5 column (150 mm × 2.1 mm, 2.6 µm; 00F-4723-AN) from Phenomenex, acylcarnitine analysis (38, 39) on a Kinetex EVO C18 column (150 mm × 2.1 mm, 2.6 µm; 00F-4725-AN) from Phenomenex, and glycerophospholipid analysis (40) via flow-injection-analysis without column. HPLC-ESI-MS/MS analyses were performed on an Agilent 1260 HPLC system comprising of a binary pump (G1312B), degasser (G1379B), and multi-sampler (G7167A) from Agilent (Waldbronn, Germany) combined with a MayLab MistraSwitch column oven from MayLab (Vienna, Austria) and a QTRAP 4000 mass spectrometer with a Turbo V ESI source from Sciex (Ontario, Canada). MS-data acquisition was performed with Analyst 1.6.3 and MS-data quantification with MultiQuant 3.0.3 from Sciex.

### Statistics and Data Analysis

The QC_CV function in the MWASTools R package (41) was used to calculate the CoV (coefficient of variation) per metabolite across QC samples (42). Preliminary analysis of the raw metabolomics data revealed significant differences in the order of magnitude of metabolite concentrations; to correct for this, normalization was performed using the hRUV (hierarchical approach to removal of unwanted variation) R package (43). We followed 2 main steps in the hRUV process: (i) adjusting signal drift within batches using robust smoothers; and (ii) correcting datasets for unwanted variations using a hierarchical RUV approach.

For all considered parameters, maximum concentrations for the tested metabolites (amino acids, organic acids, short and long-chain fatty acid carnitine esters, free carnitine) were compared between the 2 milk formulas via paired parametric or nonparametric tests. We employed linear mixed models (LMMs) to study the dynamic evolution of each metabolite over time and to identify differences between HP and LP at different time points.

To obtain the average BCAA concentrations per individual, the concentrations of leucine, isoleucine, and valine were summed for each time point, and the relationships between BCAA, their corresponding degradation products, and urea were examined. Additionally, we generated piecewise segmented regression models for the HP and LP groups independently. These models were based on the mean BCAA concentrations and may result in a regression model with an elbow function that produces a broken, discontinuous line if there are any breakpoints (44). If the *P* value falls below the significance level of .05, the breakpoints are considered statistically significant. Individual observations with noticeable impact on the estimated breakpoints were removed.

Comparisons between primary and secondary outcomes, as well as exploratory outcome measures between the 2 milk formulas were performed with paired *t* tests, or Wilcoxon rank test when deemed appropriate. Further, to test for the effects of other factors, such as BMI, fat mass index, fat mass, fat-free mass (lean mass), gender, and age on BCAA, BCKA, acylcarnitines, and urea, Pearson correlations were utilized. All statistical analyses for metabolic datasets were conducted with R statistical programming and GraphPad Prism.

## Ethics

The TOMI Add-On Study was reviewed by the ethics committee of the LMU Medical Faculty, Munich, Germany (REF 19-922). The study was conducted according to the Declaration of Helsinki and registered under the German Clinical Trial Register (No. DRKS00016169).

## Results

### Subject Characteristics

Twenty-seven subjects (15 female, 12 male) with an average age of 26.44 ± 5.06 years (mean ± SD) participated in the study; a detailed overview of baseline characteristics is provided in Newton-Tanzer et al (13).

For the 27 participants (men and women combined), an average BMI of 22.12 kg/m^2^ ± 2.46 kg/m^2^, a fat mass index (FMI) of 21.04 kg/m ± 7.01 kg/m and a fat mass (FM) of 13.85 kg ± 4.01 kg was measured. Female subjects had a BMI of 21.06 kg/m^2^ ± 2.17 kg/m^2^, with a FM of 15.02 kg ± 3.87 kg, and a FMI of 25.40 kg/m ± 5.44 kg/m. For male subjects, we observed an average BMI of 23.43 kg/m^2^ ± 2.23 kg/m^2^, a FM of 12.38 kg ± 3.83 kg, and a FMI of 15.58 kg/m ± 4.78 kg/m. Thus, the FMI was about 39% higher (*P* < .001) in female participants.

### Postprandial BCAA, BCKA, Acylcarnitine, and Urea

Fasted baseline concentrations, maximum concentration (Cmax), and time of maximum concentration (Tmax) values for BCAA, BCKA, acylcarnitines, and urea are shown in Table 1. There was hardly any increase in plasma concentration after the LP challenge in BCAA; however, the HP challenge more than doubled maximum concentrations for leucine and isoleucine. Accordingly, average concentrations were about 2-fold higher in HP vs LP for leucine and isoleucine but for valine only 37%. Maximum concentrations were generally seen later after HP than LP, except for KIVA.

**Table 1.** Fasted baseline concentrations, maximum concentration (Cmax), mean time at maximum concentration (Tmax; minutes) of BCAA (leucine, isoleucine, and valine), BCKA (KICA, KMVA, and KIVA), acylcarnitine (C5:0-3M, C5:0-2M, C4:0-iso), and urea after intake of HP and LP for the 27 subjects

BCAA (μmol/L)	HP	LP	*P* value*
Leucine			
Baseline	114 ± 26	115 ± 25	.828
Cmax	242 ± 40	133 ± 21	< .001
Tmax	77 ± 96	35 ± 59	.136
Isoleucine			
Baseline	61 ± 15	64 ± 21	.391
Cmax	141 ± 30	75 ± 18	< .001
Tmax	64 ± 87	35 ± 59	.277
Valine			
Baseline	174 ± 37	183 ± 38	.204
Cmax	284 ± 63	207 ± 39	< .001
Tmax	118 ± 112	43 ± 60	.009
**BCKA (μmol/L)**			
KICA			
Baseline	64 ± 12	55 ± 7	.837
Cmax	71 ± 12	60 ± 9	< .001
Tmax	118 ± 123	82 ± 81	.216
KMVA			
Baseline	38 ± 9	38 ± 7	.436
Cmax	71 ± 9	43 ± 7	< .001
Tmax	61 ± 68	46 ± 45	.350
KIVA			
Baseline	13 ± 4	15 ± 3	.050
Cmax	16 ± 4	18 ± 4	.075
Tmax	152 ± 120	121 ± 105	.323
**Carn (μmol/μL)**			
Carn C5:0-3M			
Baseline	.1 ± .03	.1 ± .03	.652
Cmax	.17 ± .07	.14 ± .05	< .001
Tmax	191 ± 98	108 ± 84	.002
Carn C5:0-2M			
Baseline	.04 ± .01	.04 ± .01	.953
Cmax	.05 ± .01	.04 ± .01	< .001
Tmax	168 ± 93	94 ± 63	< .001
Carn C4:0-iso			
Baseline	.1 ± .05	.1 ± .06	.121
Cmax	.08 ± .03	.06 ± .03	< .001
Tmax	211 ± 76	107 ± 72	< .001
**Urea (mg/dL)**			
Baseline	25 ± 9	27 ± 8	.287
Cmax	28 ± 8	27 ± 8	.512
Tmax	235 ± 90	23 ± 27	< .001

Abbreviations: BCAA, branched-chain amino acids; BCKA, branched-chain α-keto acids; Carn C4:0.iso, iso-butyrylcarnitine; Carn C5:0-2M, 2-methyl-valerylcarnitine; Carn C5:0-3M, 3-methyl-valerylcarnitine; HP, higher protein test meal formula; KICA, α-ketoisocaproate/4-methyl-2-oxovaleric acid/3-methyl-2-oxovaleric acid; KIVA, α-ketoisovalerate/3-methyl-2-oxobutanoic acid; KMVA, α-keto-β-methylvalerate/3-methyl-2-oxovaleric acid; LP, lower protein test meal formula.

^*^ *P* value from paired *t* test and Wilcoxon rank test for Tmax.

**Figure 1.**
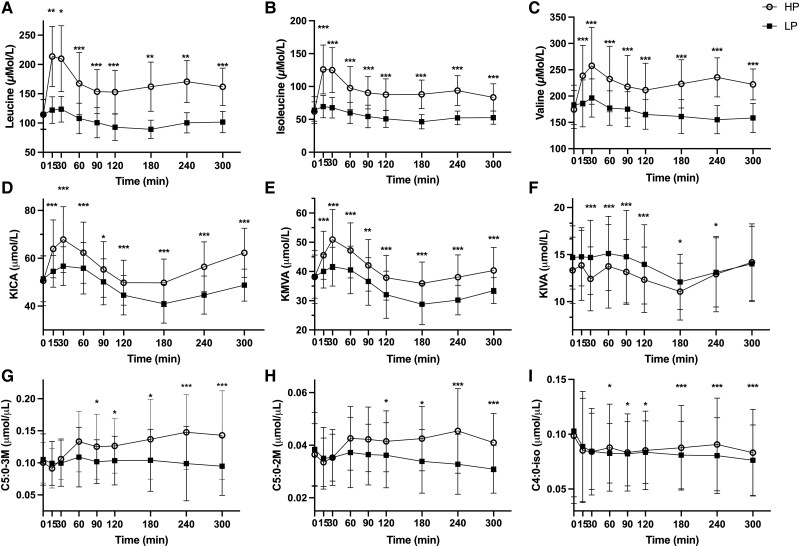
Response of serum concentrations of BCAA—leucine (A), isoleucine (B) and valine (C)—as well as their degradation products, BCKA—KICA (D), KMVA (E), and KIVA (F)—and acylcarnitines—carn C5:0-3 m (G), carn C5:0-2 m (H) and carn C4:0-iso (I)—after consumption of higher (HP) and lower protein (LP) test meals over time for 27 subjects. Mean with individual concentration curves by test meal with 95% CI (all time points except time = 0; *P* < .05= *; *P* < .01=**; *P* < 001=***).

Mean concentration curves for BCAA, BCKA, and Carn C4:0-iso, C5:0-2M, and C5:0-3M are depicted in Fig. 1. The postprandial concentration curves for BCAA after HP show an initial steep increase in concentration with a peak within the first half hour, followed by a slight decrease and plateau. The curves for KICA and KMVA followed similar patterns as the BCAA after HP consumption, showing peaks around 30 minutes postprandially, followed by a subsequent decrease until 180 minutes and then a gradual increase until the end of the study. This contrasted with the concentration curves for acylcarnitines, which, after HP, showed an initial decrease until 15 minutes, followed by an initial steeper incline until 60 minutes and then a more gradual increase until 240 minutes, followed by a decrease. Cmax and Tmax for all 3 acylcarnitines was also significantly higher after HP than LP. Interestingly, KIVA was the only metabolite to depict a postprandial concentration curve with significantly higher concentrations after LP vs HP.

### BCAA, Keto Acid, Acylcarnitine, and Urea Regression Breakpoints

**Figure 2.**
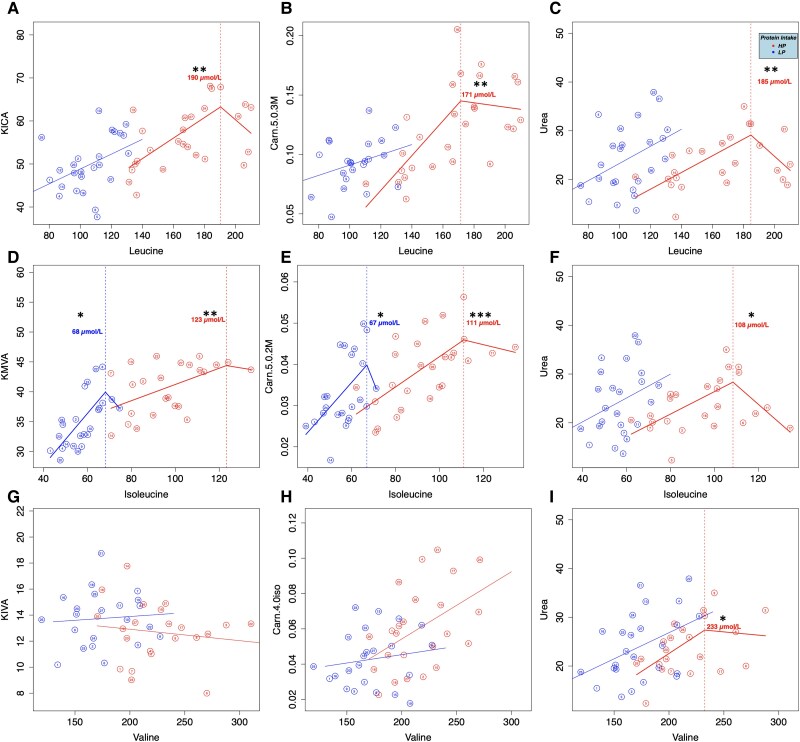
Results of the piecewise regression analysis of the BCAA degradation products and urea after the HP and LP. Metabolite concentrations averaged from all participants (solid lines) were estimated with piecewise linear mixed models, while individual means are represented by dots with the subject identification number. A, leucine, and KICA; B, leucine and C5:0-3M; C),leucine, and urea; D, isoleucine and KMVA; E, isoleucine and C5:0-2M; F, isoleucine and urea; G, valine and KIVA; H, valine and C4:0-iso; I, valine and urea. Corresponding *P* values of piecewise regression plots using segmented linear models are noted with an asterisk (*P < .05, **P < .01, ***P < .001).

The relationship between BCAA and their respective BCKA and acylcarnitine degradation products and for BCAA and urea for HP and LP is shown in Fig. 2. To observe the effects of higher and lower protein on BCAA degradation, HP and LP metabolite concentrations were grouped separately, with different breakpoint analyses. As the HP test meal contained more protein, we hypothesized that we may observe different, if not only breakpoints after HP vs LP.

A significant breakpoint for BCAA and their respective degradation products (BCKA and acylcarnitines) after HP was seen, except for valine and KIVA. Interestingly, a significant breakpoint was also observed between isoleucine and corresponding degradation products after LP: isoleucine and KMVA at 68 μmol/L, as well as isoleucine and Carn C5:0-2M at 67 μmol/L, respectively. We also observed a significantly later Tmax for valine, as well as for all 3 acylcarnitines after HP vs LP, with the Tmax for HP occurring at least an hour later than LP. However, valine and its degradation products showed the latest Tmax after HP of all analyzed metabolites.

Statistical significance of each breakpoint is noted in Fig. 2 with an asterisk corresponding to a *P* value: *P < .05, **P < .01, ***P < .001. We observed statistically significant breakpoints for leucine and isoleucine with HP, as well as 2 breakpoints for valine with HP in analysis with urea and Carn C4:0-iso. We also observed significant breakpoints for leucine and isoleucine with their respective acylcarnitines after LP.

### Effects of Age, Gender, and Anthropometry on Metabolic Response

Age, gender, and anthropometrics (weight, BMI, and FM) of participants were examined in relation to the areas under the curves (AUCs) and mean concentrations of BCAA, BCKA, and acylcarnitine. Only fat mass after HP correlated negatively and significantly with AUC BCAA and 2 BCKA, KICA, and KMVA, but not KIVA (BCAA: Leucine: r_fat mass_ = −0.42; Isoleucine: r_fat mass_ = −0.40; Valine: r_fat mass_ = −0.43 with *P ≤ .05; KICA: r_fat mass_ = −0.45; KMVA: r_fat mass_ = −0.40, with *P ≤ .05).

## Discussion

Emphasis has been placed on the unique role of BCAA in protein metabolism, particularly in the early protein hypothesis (45). Our results indicate that moderate increases in dietary protein intake may oversaturate the BCAA degradation pathway in the acute postprandial phase. While these results cannot be directly extrapolated onto children, they support both pediatric and adult studies that indicate that higher protein intake is associated with higher plasma concentrations of amino acid and insulinogenic BCAA in circulation (46, 47). This was further supported by our results, which showed hardly any increase in plasma concentration after the LP challenge in BCAA, while the HP challenge more than doubled maximum concentrations for leucine and isoleucine.

To better elucidate the mechanisms underlying this increase in circulating BCAA, we observed postprandial metabolite concentrations after intake of HP and LP separately, with the assumption that the consumption of different protein concentrations may trigger distinct metabolic states due to the saturation and/or oversaturation of specific enzymes (48). This is commonly observed in metabolic pathways where higher substrate availability can lead to altered metabolic flux and thus potentially different breakpoints in the production and/or degradation of metabolites (49). In addition, at lower protein concentrations, certain metabolic pathways might be less active or reach a maximum rate of reaction, so that higher protein may alter kinetics and lead to different breakpoints, activation thresholds, or even the saturation of pathways (50).

This hypothesis was first visible in the concentration curves of the metabolites, particularly those of BCKA and acylcarnitines after HP. The concentration curves depicted that once BCKA concentrations reached a peak at around 30 minutes postprandial, acylcarnitine concentrations appear to steadily increase until about 240 minutes. However, at this point, acylcarnitines again appear to decrease, as BCKA concentrations begin to increase again, while BCAA simultaneously gradually decrease from 240 to 300 minutes. These observations also aligned with the breakpoint analysis that showed significant breakpoints for all metabolites and their degradation products, except for valine and KIVA and valine and Carn C4:0-iso. These findings suggest a possible hierarchy in substrate affinity between BCAA, with valine possibly requiring higher concentrations to potently compete for common enzymes and transporters (LAT1 and LAT2) than leucine and isoleucine (51). BCAA share a common plasma membrane transporter, a common enzyme for the initial step of transamination BCAT, as well as oxidative decarboxylation of BCKA (BCKDH), the latter acting as both the rate-controlling and limiting step (52). Schauder et al also noted the possibility of this substrate competition, as their results depicted lower increases in KIVA concentrations after the consumption of a higher protein meal in comparison to the other 2 BCKA (53). Harper et al further showed that the Michaelis constant (Km; the substrate concentration at which the reaction rate is half of the maximum rate of reaction) of BCAT was significantly higher for valine than isoleucine or leucine in animal models (54). Hoffer et al performed additional tracer experiments in humans, depicting that leucine deamination is generally preferred over valine by a factor of 2 (55). Thus, it is possible that this acute reduction in KIVA concentration seen in Fig. 1 after HP could be due to a lower rate of catabolization (ie, transport and/or transamination) of valine, due to increased plasma concentrations of leucine and isoleucine.

However, as only leucine is solely ketogenic, these observations could also be because both isoleucine and valine cannot be fully oxidized in muscle tissue (56). BCAA oxidation takes place predominantly in skeletal muscle (65%), as opposed to the liver where most amino acids are catabolized (57); BCAA undergo both reversible transamination and irreversible oxidative decarboxylation (58, 59). Yet, these commonalities suggest that BCAA catabolism is not solely triggered by a need for glucose or ketone bodies, as muscle is not gluconeogenic but rather ketogenic tissue, highlighting potential metabolic discrepancies between BCAA based on their ketogenic and/or glucogenic status (56). To this end, animal models have illustrated that while KICA, a product of solely ketogenic leucine, is almost completely oxidized (60), KIVA, a product of solely glucogenic valine, is not (61). These observations align with the results of the present study, as KIVA depicted a much later Tmax after HP than KMVA and KIVA, suggesting that its catabolism occurs later, possibly due to a weaker affinity for the common transporter and enzymes. However, KMVA, a degradation product of isoleucine that is both gluconeogenic and ketogenic, had the earliest Tmax of BCKA, suggesting the highest substrate affinity.

To assess the correlation between these increasing BCAA concentrations and their degradation products in the postprandial phase, we utilized a breakpoint analysis first introduced by Kirchberg et al, who hypothesized that BCAA may exceed their degradation capacity (18). In their study, they analyzed BCAA and acylcarnitines, utilizing single samples from the CHOP, a trial where infants randomly received formulas with higher and lower protein for the first 12 months of life. Initial results from CHOP indicated that for blood plasma samples at 6 months of age, infants who consumed the higher protein had increased plasma BCAA concentrations in comparison to the lower protein and the control breastfed groups (62, 63). Their analysis showed increasing plasma concentrations of leucine and isoleucine, and Carn C5:0-3M and Carn C5:0-2 M until a “breakpoint” was observed, after which subsequent amino acid increases did not correspond with an acylcarnitine increase (18). These breakpoints illustrated the possibility that increased BCAA concentrations may exceed degradation capacity, essentially oversaturating the catabolic pathway after higher protein intake. These results were further supported by Humayun et al, who also utilized a breakpoint analysis to observe the effects of different amounts of protein consumption over a period of 3 months. Interestingly, their results indicated that protein increased up until a breakpoint, after which there was a plateau, suggesting that protein catabolism reached an equilibrium (64). However, as these samples were taken in a steady-state setting, over a long timeframe, it was unclear whether the results could be extrapolated onto the acute postprandial phase.

In our breakpoint analysis, we noted that increasing concentrations of degradation products, such as BCKA and acylcarnitines, correlated with increasing BCAA concentrations until a breakpoint was reached. However, after this breakpoint, the increasing concentrations of degradation products (BCKA and acylcarnitines) no longer increased with increasing levels of BCAA, but rather decreased, contrary to the other studies observing breakpoints. In addition, each of our breakpoint concentrations, except for those of valine, were slightly lower than those observed with the CHOP samples, although these differences in breakpoint concentrations and subsequent progression could arise from the differences in a steady-state vs postprandial metabolism. We also noted significant breakpoints for isoleucine and C5:0-2M and KMVA for LP, though these were the only metabolites after LP intake to depict any breakpoint. As the concentrations after LP were significantly smaller, these findings suggest that there may be an additional factor at play that is influencing the BCKDH complex and the rate of degradation. To this end, Kirchberg et al hypothesized a lower rate of degradation at elevated concentrations of BCAA due to a nonlinear relationship between urea and BCAA (18). However, while we also observed this nonlinear relationship between urea and BCAA, urea is not a degradation product specific to BCAA, but rather amino acids in general.

Limitations of this analysis are the inter- and intra-individual variability in blood metabolite concentrations. As the test meal was isocaloric with alternating higher and lower concentrations of protein and fat without any exchange between carbohydrates and protein, it was not possible to see how these metabolites may have interacted with beta-oxidation. Additionally, participants were asked to avoid physical exertion during the interventions, so that the metabolic response observed only represents and accounts for sedentary behavior. In addition, this study focused solely on the postprandial degradation of BCAA, without looking at anabolic or catabolic hormones (such as insulin and glucagon), or fatty acids and triglycerides.

Strengths of this analysis include the use of standardized test meals, as well as the uniform study conditions. As the test meal is consumed in a fasted state and observed over a longer period than other studies, it was possible to observe changes in concentrations over the time period, providing insight into metabolic variability in the acute, postprandial phase.

In conclusion, our analysis presents new evidence for saturation of the BCAA degradation pathway after higher milk protein intake in the acute postprandial phase, with significantly increased concentrations of BCAA and BCKA up to 5 hours postprandially. These observations indicate the possibility of hierarchical substrate affinity existing between BCAA for the common transporter and the rate-limiting enzyme in catabolization, BCKDH. However, as our breakpoint analysis did not depict a plateau after the breakpoint but rather a decrease, these results also suggest that protein catabolism has not yet reached a state of equilibrium, as observed in other studies observing breakpoint regression analyses.

## Data Availability

Data described in the manuscript, code book, and analytic code will be made available upon request pending application and approval.

## Clinical Trial Information

The TOMI Add-On Study is registered in the German Registry of Clinical Trials under: DRKS00016169.

## Abbreviations

BCAA: branched-chain amino acids
BCAT: branched-chain aminotransferase
BCKA: branched-chain α-keto acids
BCKDH: branched-chain alpha-ketoacid dehydrogenase complex
BMI: body mass index
Carn C4:0.iso: iso-butyrylcarnitine
Carn C5:0-2M: 2-methyl-valerylcarnitine
Carn C5:0-3M: 3-methyl-valerylcarnitine
CHOP: Childhood Obesity Project
Cmax: concentration maximum
HP: higher protein test meal formula
HPLC-ESI-MS/MS: high-performance liquid chromatography-electrospray ionization tandem mass spectrometry
KICA: α-ketoisocaproate/4-methyl-2-oxovaleric acid/3-methyl-2-oxovaleric acid
KIVA: α-ketoisovalerate/3-methyl-2-oxobutanoic acid
KMVA: α-keto-β-methylvalerate/3-methyl-2-oxovaleric acid
LP: lower protein test meal formula
Tmax: time of maximum concentration
TOMI: Toddler Milk Intervention Study trial
